# Hemoglobin A1c in Patients with Diabetes Predict Long-Term Mortality Following Coronary Artery Surgery

**DOI:** 10.3390/jcm10122739

**Published:** 2021-06-21

**Authors:** Muhammad Abu Tailakh, Shlomo-yaron Ishay, Jenan Awesat, Liat Poupko, Gidon Sahar, Victor Novack

**Affiliations:** 1 Department of Nursing, Recanati School for Community Health Professions, Faculty of Health Sciences, Ben-Gurion University of the Negev, Beer-Sheva 8410501, Israel; 2 Nursing Research Unit, Soroka University Medical Center, Beer-Sheva 84101, Israel; 3 Department of Cardiothoracic Surgery, Division of Surgical Medicine, Soroka University Medical Center, Beer-Sheva 84101, Israel; yaronishay@gmail.com (S.-y.I.); SaharG@clalit.org.il (G.S.); 4 Faculty of Health Sciences, Ben-Gurion University of the Negev, Beer-Sheva 8410501, Israel; janan.awesat@gmail.com (J.A.); liat.poupko@gmail.com (L.P.); VictorNo@clalit.org.il (V.N.); 5 Division of Internal Medicine, Soroka University Medical Center, Beer-Sheva 84101, Israel; 6 Medical School for International Health, Faculty of Health Sciences, Ben-Gurion University of the Negev, Beer-Sheva 8410501, Israel; 7 Clinical Research Center, Soroka University Medical Center, Beer-Sheva 84101, Israel

**Keywords:** diabetes mellitus, hemoglobin A1c, coronary artery bypass grafting, mortality

## Abstract

Objective: to estimate the association between preoperative hemoglobin A1c (HbA1c) levels below and above 7%, and the rate of all-cause mortality (ACM) in diabetes mellitus (DM) patients after coronary artery bypass grafting (CABG) within a ten-year follow-up period. Methods: we collected data on patient HbA1c levels that were measured up to 3 months prior to isolated CABG in consecutive patients with DM, and analyzed the rates of ACM over a median of a 5.9-year post-operative period. Results: preoperative HbA1c levels were collected in 579 DM patients. The mean HbA1c was 8.0 ± 1.7%, where 206 (35.6%) patients had an HbA1c ≤ 7% and 373 (64.4%) had an HbA1c > 7%. During the follow-up period, mortality rates were 20.4% and 28.7% in the HbA1c ≤ 7% and HbA1c > 7% groups, respectively (Kaplan-Meier estimates, log-rank *p* = 0.01). Multivariable Cox proportional hazards regression, adjusted for age, gender, smoking status, chronic obstructive pulmonary disease, hypertension, chronic renal failure, old myocardial infarction, number of coronary artery bypass surgeries, and post-operative glycemic control, showed a hazard ratio of 2.67 for long-term ACM (*p* = 0.001) in patients with HbA1c > 7%. Conclusions: DM patients with high HbA1c levels prior to CABG are at higher risk for long-term complications, especially late ACM.

## 1. Introduction

The prevalence of diabetes mellitus (DM) among patients who undergo coronary artery bypass grafting (CABG) has been estimated to be 14–37% [1,2,3], and continues to rise alongside the increasing prevalence of DM in the general population.

The American Heart Association (AHA) and the European Society of Cardiology (ESC) recommend a target of glycated hemoglobin (HbA1c) <7% as a class I recommendation [4,5]. The prevalence of poorly-controlled diabetes in CABG patients varies in the literature; research reports 50–80% of DM patients having HbA1c levels exceeding 7%. Moreover, high blood glucose is an independent predictor of cardiovascular disease, regardless of a diagnosis of DM [6,7].

The impact of poor glycemic control on post-operative outcomes is discussed extensively in the literature. A meta-analysis of 11 studies found that in four studies, early and late morbidity rates were four-fold in patients with HbA1c levels greater than 8.9% (regardless of whether the patient was diagnosed with DM before surgery), while four other studies showed no significant difference in 30-day post-operative mortality. Moreover, three studies showed a high incidence of infections in patients with uncontrolled HbA1c, while other studies in this meta-analysis found no difference in length of hospitalization [8]. However, the long-term association between HbA1C and all-cause mortality in DM patients undergoing CABG is yet to be fully elucidated.

This study aims to determine whether preoperative HbA1c values can predict long-term mortality in patients with diabetes undergoing coronary bypass surgery.

## 2. Materials and Methods

### 2.1. Study Design

We enrolled all consecutive patients undergoing their first on-pump CABG procedure between 2002 and 2013 at Soroka University Medical Center (SUMC). SUMC is the only tertiary hospital (1100 beds) in southern Israel, serving a population of 1.2 million. It is the sole provider of acute cardiac care and cardiovascular surgery in the region.

We included patients with DM who underwent isolated CABG during the study period (this study discusses complete arterial revascularization; since 2006 we routinely used bilateral internal mammary and the third conduit radial artery), and collected their demographic data, clinical characteristics, and HbA1c values.

We collected other relevant clinical variables during the follow-up period, such as hypertension, acute coronary syndrome, hyperlipidemia, chronic renal disease, smoking status, congestive heart failure, and chronic obstructive pulmonary disorder.

The study was granted approval by the hospital ethics committee (EC), EC number 0333-18-SOR.

### 2.2. Measurement of HbA1c

Blood samples obtained for measuring HbA1c levels were collected within 3 months prior to admission. The laboratory values were extracted from a centralized, regional database of patient medical records (inpatient and outpatient). All HbA1c testing was performed by a single laboratory at SUMC with Bio-Rad Variant II Turbo HbA1c Kit-2.0, based on ion-exchange high-performance liquid chromatography (HPLC) [9].

### 2.3. Diabetes Mellitus Status

Known DM status was determined by outpatient records or evidence of regular use of anti-diabetic medications.

### 2.4. Study Outcomes

The primary outcome measured was all-cause mortality (ACM) over a ten-year follow-up period. Other observed outcomes included MACCE (a composite outcome of ACM, stroke, myocardial infarction, and revascularization), immediate complications during hospitalization (including post-surgery atrial fibrillation (AF), pneumonia, cardiogenic shock, wound infection, re-surgery, and acute renal failure), and late complications during the follow-up period (including ST elevation myocardial infarction, non-ST elevation myocardial infarction, and re-hospitalization due congestive heart failure).

### 2.5. Post-Operative Glycemic Control

HbA1c values in the post-operative period were collected, calculated as median (IQR) values, and compared between study groups.

### 2.6. Statistical Analysis

Patient characteristics are presented as mean ± SD values for continuous variables and as percentage values for categorical variables. Categorical variables were compared using the chi-square test, while continuous variables were analyzed with the Student’s t-test.

The rates of adverse outcomes during follow-up (at one, two, and ten years) were estimated using the Kaplan-Meier method, with the differences between groups assessed by the log-rank test. We performed a Cox proportional hazards regression analysis to investigate the effect of DM status (categorized as HbA1c above or below 7%) on clinical outcomes in the follow-up period. To adjusted for confounders, we stratified the variables that were statistically and clinically associated with the primary outcome (ACM) into multivariable models. All clinical and demographic variables that were found by univariate analysis to differ significantly between groups (*p* < 0.10) were included in the first step of the model. Forward stepwise regression was utilized to eliminate variables that had no significant association (*p* > 0.10) with the outcome, while clinically significant variables, such as gender, smoking, hypertension, chronic renal failure, and history of MI, were forced into the models.

The hazards proportionality was assessed by the evaluation of the interaction between log (survival time) and the variable of interest. Hazard ratios (HRs) and 95% confidence intervals were estimated for each variable in the final parsimonious models.

All statistical analyses were performed using IBM SPSS version 26 (Chicago, IL, USA).

## 3. Results

### 3.1. Population

All 579 DM patients enrolled in the study had their HbA1c values measured within three months prior to undergoing CABG. Of these patients, 373 (64.4%) had HbA1c >7% and 206 (35.6%) had HbA1c below or equal to 7%. The median follow-up period was 5.9 years (IQR 2.9, 8.3).

The baseline clinical characteristics of the subjects were stratified by HbA1c levels, as presented in Table 1. Patients with HbA1c ≤7% were noted to be significantly older than those with HbA1c >7% (67.0 ± 9.0 and 63.0 ± 9.0, respectively; *p* < 0.001). No other differences were found in gender, tobacco use, or chronic illnesses such as hypertension, congestive heart failure, chronic obstructive pulmonary disease, and history of myocardial infarction.

### 3.2. Clinical Outcomes

#### Early Outcomes

Excluding post-operative AF, in-hospital complications were not significantly different between the two HbA1c groups (Table 2). Seventy-four (12.8%) patients developed post-operative AF, with 36 (17.5%) and 38 (10.2%) cases in the HbA1c ≤7% and HbA1c >7% groups, respectively (*p* = 0.01). ACM rates were higher in patients who developed post-operative AF compared to patients who did not (41.9% vs. 19.8%, respectively; Kaplan-Meier estimates log-rank *p* < 0.001).

### 3.3. Late Outcomes

**Figure 1:**
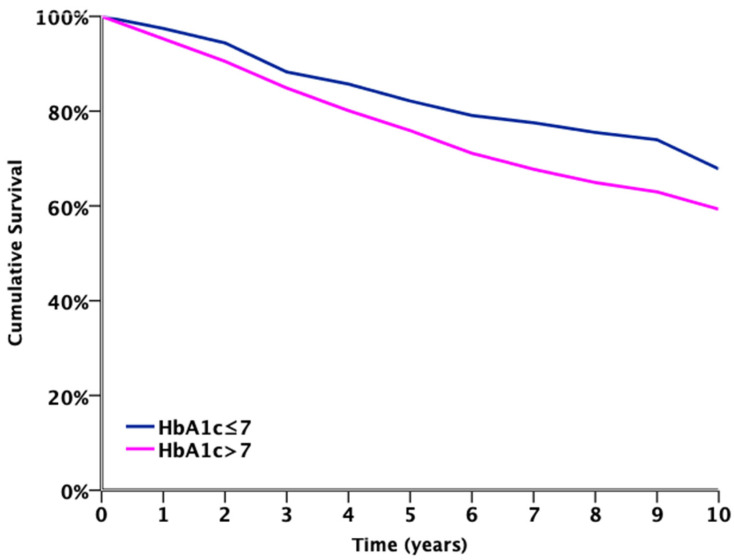
Ten-year follow-up Kaplan-Meier survival for all-cause mortality. (Log-rank *p* = 0.007).

The 10-year follow-up Kaplan-Meier survival analysis for ACM (Figure 1) estimated poor survival in patients with HbA1c >7% compared to those with HbA1c ≤7% (log-rank *p* = 0.007). The 10-year MACCE rate was significantly higher in patients with HbA1c >7%, compared to patients with HbA1c ≤7% (41.6% vs. 34.5%, respectively; *p* = 0.04), as shown in Table 3.

The Cox proportional hazards regression model was adjusted for age, gender, smoking status, chronic obstructive pulmonary disease, hypertension, chronic renal failure, history of myocardial infarction, number of bypassed coronary arteries, and post-operative glycemic control, and showed an association of HbA1c >7% with an ACM hazard ratio of 2.67 (95% CI 1.52, 4.68), as depicted in Table 4.

### 3.4. Post-Operative Glycemic Control

**Figure 2:**
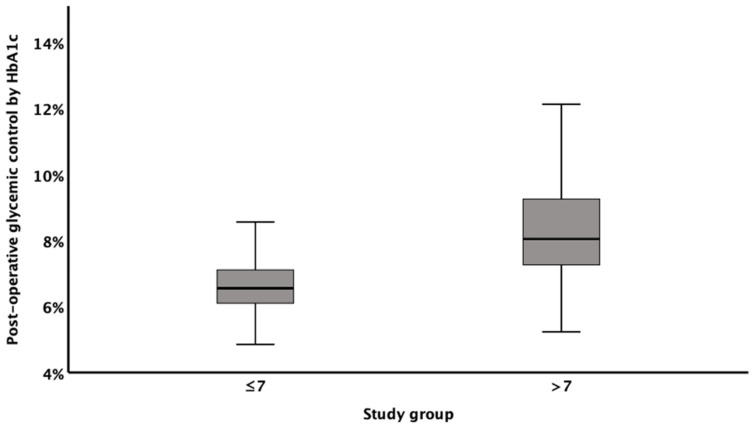
Distribution of post-operative glycemic control.

To determine the extent of glycemic control after surgery, HbA1c values from the post-operative follow-up period were compared between study groups (Figure 2). As shown, patients who underwent surgery with an HbA1c <7% had better glycemic control after CABG compared to patients with preoperative HbA1c >7% (median 6.5% vs. 8.1%, respectively; *p* < 0.001).

## 4. Discussion

In this study, almost two-thirds of the patients that had undergone CABG had poorly controlled diabetes (HbA1c > 7%). Inadequate glycemic status was associated with an increase in ten-year all-cause mortality.

Multiple studies showed that patients with diabetes are predisposed to worse short- and long-term outcomes after CABG, relative to patients without diabetes mellitus (DM) [10]. Preoperative HbA1c has been previously identified as a clinical indicator for quantifying an individual’s risk for post-CABG complications [8,11,12]. However, no study in the literature has conducted a follow-up on these patients beyond a five-year period.

In the present study, after adjustment for several known risk factors, patients with HbA1c >7% had a significantly increased risk of long-term mortality. Considering this finding, it is of essence to determine whether candidates for CABG should achieve better glycemic control prior to surgery even at the cost of delaying elective surgery.

Reports are conflicting as to whether high preoperative HbA1c is associated with post-operative complications or mortality in patients with DM [3,7,11,13]. Consistent with our results, a prospective study with a 3.5-year average follow-up by Alserius et al. found an 18.9% incidence of mortality with preoperative HbA1c ≥6%, compared to 4.1% in patients with HbA1c <6% [14]. In a similar vein, Halkos et al. observed lower 5-year survival rates in patients with HbA1c ≥7%, compared to patients with HbA1c <7% [11]. The authors proposed that preoperative optimization of HbA1c may improve long-term survival and that HbA1c is a potential reliable marker for long-term morbidity in patients with a history of CABG. The major factors that influence these outcomes are the severity of diabetes-associated comorbidities and end organ complications, such as renal insufficiency and peripheral vascular disease.

On the other hand, Tsuruta et al. reported, in a study of 893 patients who underwent off-pump CABG, that preoperative HbA1c levels may not be a reliable predictor of long-term outcomes. However, it should be noted that post-operative HbA1c levels in those subjects were not documented [15], while the present study adjusted for post-operative glycemic control as a potential confounding factor for late mortality.

The association between elevated pre-CABG HbA1c and the occurrence of post-operative atrial fibrillation (AF) is also under debate. Although some research reports a reduction of AF in those with elevated pre-operative HbA1c, others suggest the opposite. In line with our results, Kinoshita et al. found in a study of 805 patients that higher HbA1c levels were independently associated with a lower incidence of AF in the post-operative period [16]. Notably, in contrast with the present study, those patients underwent off-pump surgery. Again, in a study of 3089 patients undergoing elective primary coronary surgery, Halkos et al. found a 20.9% incidence of AF in patients with HbA1c of <7% and 15.1% in patients with HbA1c ≥7% (*p* = 0.007). One possible explanation for the lower AF incidence in the present study is that the patients with higher HbA1c were significantly younger than those with lower HbA1c.

As in the literature [17], the potential clinical relevance of our study pertains to the use of an HbA1c threshold of 7% for predicting the risk of adverse outcomes after CABG. Physicians may find this marker useful as an adjunct to their clinical discretion when determining whether their patient’s glycemic status should be corrected before surgery.

### Limitations

One limitation of this study was the lack of data on the effects of medications that were more recently introduced to the market, which may have been added to the patients’ regimens after surgery. Such treatments may have impacted long-term cardiovascular outcomes. During the follow-up period, we determined the extent of glycemic control through repeated HbA1c measurements and adjusted the final model accordingly.

Another limitation was that the study was conducted at a single-center; however, the population of the medical center is considerably large and diverse, serving over one million people of varied ethnicities and socioeconomic status.

The final limitation was that retrospective data collection could be incomplete, leaving out relevant clinical data that may have had an impact on patient outcomes (e.g., BMI and lipids).

We strongly recommend research on this topic to be conducted as a prospective study.

## 5. Conclusions

Patients with diabetes undergoing coronary artery bypass grafting surgery with high preoperative HbA1c levels face a higher risk of long-term complications, particularly late all-cause mortality. In the present study, the findings suggested that pre-operative diabetes control can lead to better outcomes in patients undergoing CABG, but to validate these data optimally, a prospective study should be considered. In addition, we recommend that physicians maintain closer follow-up of patients who underwent CABG before achieving adequate glycemic control.

## Figures and Tables

**Table:** 

**Number Exposed to Risk**											
**HbA1c ≤ 7**	198	196	189	181	172	166	159	153	150	146	76
**HbA1c > 7**	362	355	336	318	299	282	267	251	240	229	116
**Years**	0	1	2	3	4	5	6	7	8	9	10

**Table 1:** Baseline demographic and clinical characteristics.

Characteristics	HbA1c ≤ 7%	HbA1c > 7%	*p* Value
	*n* = 206	*n* = 373	
Age Mean ± SD	67 ± 9	63 ± 9	<0.001
Male	159 (77.2)	268 (71.2)	0.16
HTN	144 (69.9)	258 (69.2)	0.85
CHF	9 (4.4)	16 (4.3)	0.96
CRF	11 (5.3)	17 (4.6)	0.67
COPD	8 (3.9)	25 (6.7)	0.16
History of MI	56 (27.2)	88 (23.6)	0.34
Smoking	63 (30.6)	135 (36.2)	0.17

Plus–minus values are mean ± SD values. Number (percent). HTN = hypertension, CHF = congestive heart failure, CRF = chronic renal failure, COPD = chronic obstructive pulmonary disease, MI = myocardial infarction.

**Table 2:** In-hospital complications.

Characteristics	HbA1c ≤ 7%	HbA1c > 7%	All	*p* Value
	*n* = 206	*n* = 373		
MACCE	15 (7.3)	18 (4.8)	33 (5.7)	0.22
Mortality	8 (3.9)	11 (2.9)	19 (3.3)	0.55
PCI	0 (0)	0 (0)	0 (0)	---
Stroke	2 (1.0)	2 (0.5)	4 (0.7)	0.56
AMI	6 (2.9)	7 (1.9)	13 (2.2)	0.42
STEMI	1 (0.5)	2 (0.5)	3 (0.5)	0.94
NSTEMI	6 (2.9)	6 (1.6)	12 (2.1)	0.29
Atrial fibrillation	36 (17.5)	38 (10.2)	74 (12.8)	0.01
Wound infections	11 (5.3)	30 (8.0)	41 (7.1)	0.23
Acute renal failure	28 (13.6)	41 (11.0)	69 (11.9)	0.35
Cardiogenic shock	1 (0.5)	5 (1.3)	6 (1.0)	0.33
Re-surgery due to post-operative bleeding	7 (3.4)	11 (2.9)	18 (3.1)	0.76
Pneumonia	4 (1.9)	10 (2.7)	14 (2.4)	0.58
ACS	7 (3.4)	8 (2.1)	15 (2.6)	0.36
Median length of stay (IQR)	6 (4; 8)	6 (4; 8)	6 (4; 8)	0.58

Number (percent). PCI = percutaneous coronary intervention, AMI = acute myocardial infarction, STEMI = ST elevation myocardial infarction, NSTEMI = non-ST elevation myocardial infarction, ACS = acute coronary syndrome, MACCE = major adverse cardiovascular and cerebrovascular events (defined as all-cause mortality, stroke, myocardial infarction, or revascularization).

**Table 3:** Ten-year post-operative complications, Kaplan-Meier estimation.

Characteristics	HbA1c ≤ 7%	HbA1c > 7%	All	*p* Value
	*n* = 206	*n* = 373		
MACCE	71 (34.5)	155 (41.6)	226 (39.0)	0.04
Mortality	42 (20.4)	107 (28.7)	149 (25.7)	0.01
AMI	26 (12.6)	46 (12.3)	72 (12.4)	0.76
-STEMI ^	2 (1.0)	3 (0.8)	5 (0.9)	0.09
-NSTEMI ^	25 (12.1)	44 (11.8)	69 (11.9)	0.78
PCI	24 (11.7)	40 (10.7)	64 (11.1)	0.97
Stroke	10 (4.9)	29 (7.8)	39 (6.7)	0.13
CHF hospitalization	4 (1.9)	6 (1.6)	10 (1.7)	0.89
Glycemic control by HbA1c% Median (IQR)	6.5 (6.1; 7.1)	8.1 (7.3; 9.2)	7.5 (6.6; 8.5)	<0.001

Number (percent). STEMI = ST-elevation myocardial Infarction, NSTEMI = non-ST elevation myocardial infarction, PCI = percutaneous coronary intervention, CHF = congestive heart failure, AMI = acute myocardial infarction, MACCE = major adverse cardiovascular and cerebrovascular events (defined as all-cause mortality, stroke, myocardial infarction, or revascularization). Glycemic control by HbA1c% Median (IQR): all values of HbA1c in follow-up period (post-operative) presented as median and interquartile range. ^ same pathology of AMI.

**Table 4:** Ten-year follow-up, Cox regression analysis for ACM.

Characteristics	Hazard Ratio	*p* Value	95% Confidence Interval	
			Lower	Upper
Age (Years)	1.04	0.01	1.01	1.06
Old MI	1.87	0.01	1.19	2.92
History of CHF	2.20	0.06	0.96	4.93
CRF	4.49	<0.001	2.11	9.54
COPD	2.60	0.01	1.28	5.28
HbA1c > 7%	2.67	0.001	1.52	4.68
Glycemic control	1.003	0.97	0.86	1.18

Data were adjusted for gender, smoking, hypertension, and number of coronary artery bypass procedures. Old MI = old myocardial infarction, CRF = chronic renal failure, COPD = chronic obstructive pulmonary disease, HbA1c = hemoglobin A1c (binary variable, >7% and ≤7%). Glycemic control: all values of post-operative HbA1c levels in follow-up period are presented as continuous values.

## Data Availability

The following data can be partially available upon the request under national regulations for data sharing.
